# Evidencing How Experience and Problem Format Affect Probabilistic Reasoning Through Interaction Analysis

**DOI:** 10.3389/fpsyg.2019.01548

**Published:** 2019-07-04

**Authors:** Manuele Reani, Alan Davies, Niels Peek, Caroline Jay

**Affiliations:** ^1^School of Computer Science, University of Manchester, Manchester, United Kingdom; ^2^Division of Informatics, Imaging and Data Sciences, University of Manchester, Manchester, United Kingdom

**Keywords:** probabilistic reasoning, interaction analysis, bayesian inference, decision making, mouse movement

## Abstract

This paper examines the role that *lived experience* plays in the human capacity to reason about uncertainty. Previous research shows that people are more likely to provide accurate responses in Bayesian tasks when the data are presented in natural frequencies, the problem in question describes a familiar event, and the values of the data are in line with beliefs. Precisely why these factors are important remains open to debate. We elucidate the issue in two ways. Firstly, we hypothesize that in a task that requires people to reason about conditional probabilities, they are more likely to respond accurately when the values of the problem reflect their own lived experience, than when they reflect the experience of the average participant. Secondly, to gain further understanding of the underlying reasoning process, we employ a novel interaction analysis method that tracks mouse movements in an interactive web application and applies transition analysis to model how the approach to reasoning differs depending on whether data are presented using percentages or natural frequencies. We find (1) that the closer the values of the data in the problem are to people's self-reported lived experience, the more likely they are to provide a correct answer, and (2) that the reasoning process employed when data are presented using natural frequencies is qualitatively different to that employed when data are presented using percentages. The results indicate that the benefits of natural frequency presentation are due to a clearer representation of the relationship between sets and that the *prior* humans acquire through experience has an overwhelming influence on their ability to reason about uncertainty.

## 1. Introduction

Over the past five decades, the human ability to reason about uncertainty has been the subject of a wealth of research. A large amount of evidence has shown that humans struggle with certain forms of probabilistic reasoning. Of particular difficulty are problems where one is expected to use Bayes' theorem (Equation 1) to estimate the probability of a hypothesis given the availability of certain evidence. These appear to be challenging not only for laypeople but also for experts, such as medical professionals. Consider this example from an early study (Eddy, 1982):

The probability of having breast cancer for a woman of a particular age group is 1%. The probability that a woman with breast cancer will have a positive mammography is 80%. The probability that a woman without breast cancer will also have a positive mammography is 9.6%. What is the probability that a woman with a positive mammography actually has breast cancer?

To answer the question one should apply Equation 1 in which P(H ∣ E), known as the posterior probability or the positive predictive value (PPV), is the probability of the hypothesis (breast cancer) given the evidence (positive mammography), P(E ∣ H), known as the likelihood or sensitivity of the test, is the probability of the evidence given the hypothesis, P(H), known as the prior probability or base rate, is the probability of the hypothesis, P(E ∣ ¬H), known as the false positive rate or false alarm rate, is the probability of the evidence given the opposite hypothesis (e.g., no breast cancer) and P(¬H) is the probability of the opposite hypothesis.

(1) $$\begin{array}{l} P\left(H|E\right)=\frac{P\left(E|H\right)P\left(H\right)}{P\left(E|H\right)P\left(H\right)+P\left(E|\neg H\right)P\left(\neg H\right)} \end{array}$$

The answer to this problem in the original paper, achieved by applying the equation to the figures given in the question, is 7.8%. When posed to a group of physicians, however, only around 5% of them arrived at the correct estimate; the majority estimated a probability of between 70 and 80% (Eddy, 1982). Many subsequent studies have reported similar results, and for at least four decades there has been an ongoing debate about why people perform so poorly in probabilistic reasoning tasks (McDowell and Jacobs, 2017; Weber et al., 2018). Among the many explanations given, two have been reported extensively in previous literature. One theory is that many people fail to make a correct inference because they do not adequately consider the base rate—a phenomenon known as base rate neglect (Tversky and Kahneman, 1974; Bar-Hillel, 1983). When the base rate value is very small, this can lead to a large overestimation of the PPV, as found in the mammography problem study (Eddy, 1982). A second theory is that people who fail to make a correct inference confuse the sensitivity, i.e., P(E ∣ H), with the PPV, i.e., P(H ∣ E) (Eddy, 1982; Elstein, 1988; Gigerenzer and Hoffrage, 1995; Gigerenzer et al., 1998; Hoffrage and Gigerenzer, 1998). Previous research suggests that there are other factors affecting probabilistic reasoning. The information format in which the problem is described appears to be strongly linked to how people perceive probabilistic problems (Gigerenzer and Hoffrage, 1995; Binder et al., 2018). Furthermore, people's beliefs about the uncertainty surrounding the event described in the problem (which may be the result of direct experience) can also affect how they perceive and reason about probabilities (Cohen et al., 2017). At present, however, the cognitive processes involved in this form of reasoning remain poorly understood, and a full account of how these factors affect reasoning is still lacking (Weber et al., 2018). The current study has two aims. The first is to examine whether the previous *lived experience* people have with the uncertainty surrounding a real-life stochastic event affects their reasoning about the probability of such an event. We hypothesize that personal beliefs about uncertainty formed as a result of lived experience, reinforced over time, can bias people's estimation of risk. A second aim of the study is to investigate whether the format in which the data is presented (i.e., probabilities vs. frequencies) affects *the way people approach the problem* and whether behavioral patterns associated with the different formats can explain people's reasoning. To achieve this, we use a paradigm where information remains hidden until it is hovered over with a mouse. By tracking mouse movements, we can determine when and in what order people access the problem data, providing a window on the cognitive process.

### 1.1. Two Theories of Probabilistic Reasoning

It has been hypothesized that people's inability to answer probabilistic reasoning problems correctly might be related to the way these problems are framed, i.e., the information format (Gigerenzer and Hoffrage, 1995). The *ecological rationality framework* argues that the use of natural frequencies, or visualizations that highlight frequencies, improves probabilistic reasoning because this way of representing the problem reflects what humans have encountered in real-life situations over thousands of years of evolution (McDowell and Jacobs, 2017). The mammography problem re-framed using frequencies states:

*100 out of 10,000 women of a particular age group who participate in routine screening have breast cancer. 80 out of 100 women who participate in routine screening and have breast cancer will have a positive mammography. 950 out of 9,900 women who participate in routine screening and have no breast cancer will also have a positive mammography. How many of the women who have participated in routine screening and received a positive mammography actually have breast cancer? (Gigerenzer and Hoffrage*, *1995**)*

In this case, the calculation required to correctly answer the problem is simpler, as it reduces to dividing the number of women who have breast cancer and tested positive (80) by the number of women who tested positive regardless of whether they actually have the disease (80 + 950).

Previous research shows that the use of the frequency format, or graphs highlighting frequencies, boosts performance (Gigerenzer and Hoffrage, 1995; McDowell and Jacobs, 2017). Nevertheless, even when re-framing the problem using natural frequencies, evidence from more than 20 years of probabilistic reasoning research shows that about 76% of people still make incorrect estimates (McDowell and Jacobs, 2017). To date, it is still not clear why this is the case (Weber et al., 2018).

It is worth noting that, in this study, by “frequency format” we mean the numerical format describing a Bayesian problems where the data is presented using natural frequencies and the question asks the participant to state the frequency of events in the form of *X* out of *Y*. By “probability format” we mean the numerical format describing a Bayesian problem where the data are shown using probabilities (or percentages) and the question asks for a single-event probability. This clarification is needed as there are hybrid possibilities where, the question in a problem framed using natural frequencies can be asked as a single event probability. In this situation, the advantage of using natural frequencies appears to be diminished (Cosmides and Tooby, 1996; Tubau et al., 2018).

As shown in the above calculation, the frequency format is less computationally demanding than the probability format. According to the proponents of the ecological rationality framework, this is the main, albeit not the only reason why people reason better with frequencies. The frequency format is also argued to be more congruent with the way people acquire information in the wild (Gigerenzer and Hoffrage, 1995, 2007; McDowell and Jacobs, 2017). A strict interpretation of this framework assumes that frequencies are better processed by the human mind, as this way of representing uncertainty might be the ideal input for a cognitive mechanism specifically evolved through human phylogenesis to deal with frequencies, a position which has been challenged by some (Sirota and Juanchich, 2011; Lesage et al., 2013; Gigerenzer, 2015; Hoffrage et al., 2015; Sirota et al., 2015; McDowell and Jacobs, 2017).

A second perspective, the *nested-set hypothesis*, states that the frequency format, and related visual aids, are effective because they clearly expose relationships between sets that are not apparent when the problem is described using the probability version of the textual format (McDowell and Jacobs, 2017). According to this theory, it is less the case that the format taps into a specially evolved cognitive module, but rather that it better supports domain-general human cognition via a clearer problem presentation (Cosmides and Tooby, 1996, 2008; Sirota et al., 2015). This latter view has been supported in a number of studies (Sirota and Juanchich, 2011; Lesage et al., 2013;Sirota et al., 2015).

Some researchers hold the views that the ecological rationality framework and the nested-set hypothesis diverge in their explanation of how humans perform probabilistic reasoning, others disagree that the theories are dichotomous, stating that both explanations converge on the conclusion that the format provides an information structure that simplifies computations (McDowell and Jacobs, 2017). Furthermore, it is worth noting that the theorists who developed the ecological rationality framework had stated in their research that natural frequencies simplify the calculation because they provide a clearer structure of the problem. Thus, although they did not call this the nested-set hypothesis, it appears clear that they referred to the same concept (Hoffrage et al., 2002; Gigerenzer and Hoffrage, 2007). Although it can be argued that the two theories are in reality one, the cognitive process by which this facilitative effect is achieved is still under investigation. The present lack of consensus, and the heterogeneity found in the results of previous studies, suggest that the cognitive mechanisms underpinning how people approach probabilistic reasoning problems are still not fully understood (McDowell and Jacobs, 2017).

### 1.2. The Role of the Data Acquisition Process

The format in which information is displayed is not the only factor affecting probabilistic reasoning. Previous research suggests that the way in which people take on board information and learn probabilities – termed the *data acquisition process* – can also affect reasoning (Hoffrage et al., 2015; Traczyk et al., 2019).

Research in probabilistic reasoning is historically divided into two different families of tasks: in one case probabilities are derived from sequential experimentation; in the other probabilities are fully stated in a single instance (Hoffrage et al., 2015). Data acquisition is thus accomplished either by obtaining information through sequential experimentation, enabling a reconstruction of the likelihood, i.e., P(E ∣ H), as described in the “bags-and-chips” problem below, or by receiving an explicit statement of the likelihood and the false positive rate values, as found in the mammography problem described earlier. Early research in probabilistic reasoning was pioneered by Edwards (1968), who conducted several studies using the famous “bags-and-chips” problem (Phillips and Edwards, 1966; Edwards, 1968, 1982; Slovic and Lichtenstein, 1971). In this problem, participants are told that there are two bags filled with poker chips. One bag has 70 red chips and 30 blue chips, while the other bag has 30 red chips and 70 blue chips. Participants do not know which bag is which. The experimenter flips a coin to choose one of the bags, and then begins to randomly sample chips from the chosen bag, with replacement. Thus, before drawing any chip, each bag is equally likely to be chosen (i.e., *p* = 0.5). At the end of the sampling process, participants are left with a sequence of chips drawn from the bag, e.g., six red and four blue chips. Participants are then asked to estimate the probability that the predominantly red bag is the one being sampled. Applying Bayes' theorem to a situation where six red and four blue chips are sampled, the probability that the predominantly red bag is the one being sampled is 0.85. Several experiments using this task show that participants' estimates tend to be very close to correct, but are slightly conservative (i.e., participants have the tendency to slightly underestimate the probability that the bag chosen is the predominantly red bag) (Phillips and Edwards, 1966,?; Edwards, 1968, 1982; Slovic and Lichtenstein, 1971). Edwards and colleagues concluded that people reason in accordance with Bayes' rule, but they are “conservative Bayesians”, as they do not fully update their prior beliefs in light of new evidence as strongly as Bayes' rule prescribes (Phillips and Edwards, 1966;Edwards, 1968).

The key difference between the mammography problem and the bags-and-chips problem is that in the former, the likelihood and the false positive rate values are explicitly stated in the description of the problem; conversely, in the latter, participants have to update their beliefs sequentially, upon the acquisition of new information – i.e., the information acquisition process is staged, and subjects learn about each case serially through lived experience (Edwards, 1968; Mandel, 2014). Thus, the method used by Edwards for testing probabilistic reasoning is conceptually very different to that used in more recent research where different versions of the mammography problem have been employed. The results from previous research show that the outcomes produced by these two classes of experiments, in terms of participants' performance, are also different. In the bags-and-chips problem people's estimates, albeit conservative, tend to be fairly accurate. Conversely, the results from research using descriptive tasks (e.g., the mammography problem) have shown that people perform poorly at probabilistic reasoning and tend to greatly overestimate risk (Eddy, 1982; Gigerenzer and Hoffrage, 1995). A clear distinction can thus be made between *the probability learning paradigm*, which uses tasks in which people learn probabilities through a direct (lived) experience with the sampling process (i.e., the data acquisition process involves continuously updating beliefs over time in light of new evidence) and *the textbook paradigm* in which the probabilities are fully stated in a text or in a graph (i.e., the data acquisition process is indirect, and the temporal component is missing) (Hoffrage et al., 2015). This distinction draws a parallel with some literature in the field of decision making which highlighted a difference between decisions derived from experience and decisions from descriptions (Hertwig et al., 2004).

### 1.3. How Does Data Acquisition Affect Cognition

The probability learning paradigm employs tasks where people are given the opportunity to learn probabilities from a sequence of events, and are subsequently tested as to whether they make judgments consistent with Bayes' rule. In such tasks, performance tends to be accurate. The superior performance observed in the probability learning paradigm is hypothesized to be due to the fact that in these situations people may use unconscious, less computationally demanding (evolutionary purposeful) mental processes (Gigerenzer, 2015).

The textbook paradigm employs tasks where probabilities are numerically stated, in either a textual description or a graphical representation of the problem. People perform poorly in these tasks, particularly when the information is provided in probabilities. This effect may be due to a heavy reliance on consciously analytical (biologically secondary) mental processes that require much greater cognitive effort (Gigerenzer, 2015).

It thus appears that direct experience with uncertainty (typical of those tasks found in the probability learning paradigm) taps into statistical intuition. Conversely, descriptions that are merely abstractions of reality are not able to fully substitute for an individual's direct experience with the environment and may require (explicit) analytic thinking (Hertwig et al., 2018).

Although experience and description are different ways of learning about uncertainty, they can be complementary. Description learning may be useful when we do not have the opportunity to directly experience reality, as may be the case when events are rare, samples are small, or when the causal structure of experience is too complex (Hertwig et al., 2018). Learning on the basis of a description may also be perceived as an experiential episode, if the format of the description is able to trigger an experience-like learning process. For example, presenting a textbook problem, such as the mammography problem, in terms of natural frequencies rather than conditional probabilities, may make this task (at the perceptual level) closer to learning from experience. This would occur if frequencies from natural sampling are seen as abstractions representing the process of sequentially observing one event after the other in the real world (Hoffrage et al., 2015). If this is the case, the manipulation of the information format would affect *the perception* of the data acquisition process. This may be the reason why the proportion of people who reason in accordance with Bayes' rule rises substantially when the information is presented using natural frequencies (Gigerenzer, 2015; Hertwig et al., 2018). Nevertheless, it may also be that frequency formats are effective merely due to their ability to highlight hidden relationships (i.e., this would enable the formation of clearer mental representations of the problem) or the fact that computing the solution when the problem is framed using the frequency format is much simpler than computing the solution when the problem is framed using the probability format due to the reduced number of algebraic calculations in the former (Sirota and Juanchich, 2011; Lesage et al., 2013; Sirota et al., 2015).

### 1.4. The Role of Task Familiarity and Personal Beliefs

In probabilistic reasoning research using the textbook paradigm, people appear to be more accurate when reasoning about familiar tasks (everyday problems) than unfamiliar tasks (e.g., diagnostic medical testing) (Binder et al., 2015). There is also evidence that the degree of belief a participant has about the probability of an event affects his or her performance (Cohen et al., 2017). This latter stream of research collected people's opinions, via surveys, about the uncertainty surrounding certain stochastic events – i.e., whether the probabilities used in problems are believable or not – and subsequently tested participants on these, to show that accuracy improves when the probabilities are rated as more believable.

A person's beliefs might be formed as a result of indirect experience (e.g., a friend's story, anecdotes, news, social media, discussion forums, etc.) or from lived experience, through direct exposure to the uncertainty surrounding an event, perhaps reinforced over time (e.g., a physician dealing with mammography tests daily). Thus, the quality of one's beliefs can be the result of the way he/she acquire the information (i.e, the data acquisition process) in such problems. This draws parallel with the distinction which was made between reasoning from description and reasoning from experience presented in previous studies (Hoffrage et al., 2015). According to this line of argument, if data in a reasoning task matches beliefs emerging from lived (direct) experience of the uncertainty related to the stochastic event, people may perform better than they would if the data are simply generally plausible, and that this may hold regardless of the format in which uncertainty is encoded.

### 1.5. Rationale and Research Hypotheses

In this study, we investigate the effect of *lived experience* on reasoning accuracy. Previous research has shown that people are more accurate in their reasoning when presented with believable data, as determined at a population level (Cohen et al., 2017). There is also evidence from the experiential learning paradigm that direct experience with the data facilitates reasoning (Edwards, 1968). Indeed, some research has shown that the way in which people gather information about uncertainty affects reasoning (Hoffrage et al., 2015; Traczyk et al., 2019). We thus hypothesize (H1) that people are more likely to reason accurately when the data presented in a reasoning problem directly match their self-reported experience of the probability of an event, than when the data are believable, but do not match their experience. This is because experience-matched data may tap into those unconscious processes typically involved in experiential learning (Gigerenzer, 2015).

The second hypothesis (H2) tests whether the frequency format is superior to the probability format *only* because it resembles the process of learning from experience. The ecological rationality framework states that people reason more accurately when using the frequency format because it induces experiential learning at the perceptual level. However, when the data is derived from people' lived experience, an experiential learning process had already took place. At this point, the facilitative effect of the frequency format might be redundant. We therefore hypothesize that when data match experience, there will be no facilitative effect of presenting the problem in the frequency format, but when data do not directly match experience, this effect will be present.

Previous research using *interaction analysis* to study probabilistic reasoning has found patterns in people's observable behavior to be linked to certain reasoning strategies (Khan et al., 2015; Reani et al., 2018b, 2019). To date, this work has focused primarily on eye tracking analysis, which may not provide a comprehensive picture of an individual's reasoning process. For instance, people may fixate on certain locations not because they consciously intend to acquire the information contained in those locations, but because the physical properties of these (e.g., color, shape, etc.) attract visual attention.

In this study we therefore seek to shed further light on the reasoning processes with an online method that uses mouse-event analysis to study human cognition. In an interactive web application, the user has to hover the mouse cursor over the nodes in a tree diagram to uncover hidden information. When the mouse moves away, the information is hidden again, so it is clear when the user is accessing the data. As the relevant information is obscured by buttons, and participants must explicitly hover over the button to reveal the data underneath, it is possible to obtain a *direct link* between cursor behavior and cognition. Mouse events are then analyzed using a transition comparison method previously applied to eye tracking data (Reani et al., 2018b, 2019; Schulte-Mecklenbeck et al., 2019). We hypothesize (H3) that if probability reasoning and frequency reasoning invoke different cognitive processes, mouse movement will differ according to the format in which the information is encoded.

## 2. Method

In the mammography problem (Eddy, 1982), the jargon and the problem context may be unfamiliar to most people and, consequently, participants may not fully understand what the results of a diagnostic test actually represent in terms of risk. Previous research has shown that people are better at solving problems which are familiar to them from everyday experience (Binder et al., 2015). People are seldom exposed to diagnostic tests in every day life, unless they are medical professionals. Thus, the general public may not be able to make full use of their previous experience to evaluate uncertainty about an event, if their experience regarding this event is limited.

As a result, in this study, a “fire-and-alarm” scenario was used as a situation that is meaningful to most people (see the Supplementary Materials for the full textual description of the problem). In this context, by analogy with the mammography problem, the diagnostic test is the fire alarm, which can sound or not sound, and the disease is the fire which can be present or absent. It is very likely that participants have been exposed to at least some situations in which they have heard a fire alarm, for instance in a school or a workplace. This scenario is thus presumed to be more familiar to people than scenarios describing medical diagnostic tests, and uses simpler terminology. However, although the context of the problem is different, the information provided in the fire-and-alarm scenario is similar to the information provided in the original mammography problem, i.e., they both include the base rate (here, the probability of being in the presence of fire in a random school on a random day of the year), the true positive rate (the probability of hearing a fire alarm given that there is a real fire in the school) and the false alarm rate (the probability of hearing a fire alarm given that there is not a fire in the school).

**Figure 1:**
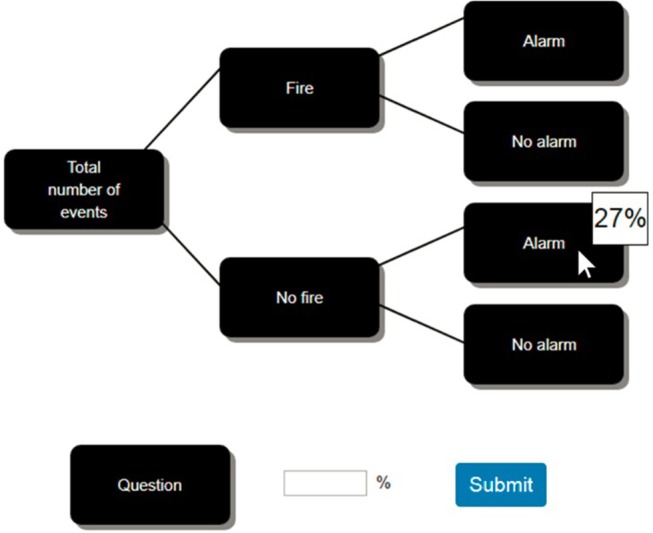
Problem shown using a tree diagram with the probability format, where the information is hidden behind the buttons, and hovering the mouse cursor over a button reveals the information underneath.

The problem was presented using a tree diagram (see Figure 1). We chose to use a graph because this clearly separates the data of the problem in space and, consequently, can be easily used to study interaction events. Bayesian problems of this kind are known to be hard to solve (Eddy, 1982), and previous research in probabilistic reasoning has used trees extensively as a clear and familiar way to display probabilistic problems (Binder et al., 2015; Hoffrage et al., 2015; Reani et al., 2018a). Some studies have shown that performance in probabilistic reasoning tasks improves when these are presented using tree diagrams containing natural frequencies, but not when these diagrams display probabilities (Binder et al., 2015, 2018). A graph can be presented alone or in conjunction with a textual description of the problem. As previous work has demonstrated that adding a textual description to a graph which already displays all the data is unnecessary and does not improve participants' performance (Sweller, 2003; Mayer, 2005; Micallef et al., 2012; Böcherer-Linder and Eichler, 2017; Binder et al., 2018), in the present research we use a tree diagram without a description of the problem. We compare frequency trees with probability trees to test our hypothesis (H2) that the manipulation of the information format does not have an effect on performance in a descriptive task which is perceived to be like an experiential learning task (details below).

Before presenting the problem, participants were given some contextual information (provided in the Supplementary Materials) which described several plausible situations that they were likely to have encountered; for instance situations in which there was a fire in a school but the fire alarm did not sound, perhaps because it was faulty, or situations in which one could hear a fire alarm but there was no fire, for instance, because someone was smoking in the bathroom. This type of contextual information is similar to the information given in the narratives used in previous experiments to reduce the artificiality of the experimental setting and improve the clarity of the problem (Ottley et al., 2016). In this case, it was also used to better relate the problem to participants' previous experience.

To investigate the effect of the data acquisition process on people's reasoning about uncertainty, two separate but comparable online studies were conducted. The data from the two studies are evaluated within the same analysis (using a between-subjects approach), as the only difference between them was the way in which the information provided in the graph was generated (the variable DGM—Data Generating Mode).

In both studies, participants were asked in a preliminary survey to provide estimates, based on self-reported experience, of the probability of fire in a given school on a random day of the year (the base rate information), the probability of hearing a fire alarm given that there was a real fire (the true positive rate) and the probability of hearing a fire alarm given that there was not a real fire (the false alarm rate). In both studies, participants were asked to provide these quantities either in the form of frequency (e.g., 2 out of 50) for the first condition, or in the form of percentages (e.g., 4%) for the second condition.

### 2.1. Study 1

In the first study, participants were shown a tree diagram displaying information derived from the values provided by the participants themselves (i.e., their self-reported experience with regard to the base rate, true positive rate and false alarm rate). To achieve this, the inputs provided by the participants during the preliminary survey (see Supplementary Materials) were stored in the Web application database, and then utilized to construct the tree that was displayed in the second phase of the task.

The study used a between-subjects design with one factor, Information Format, with two levels (frequency vs. probability). Participants were asked to provide the three quantities in the form of either natural frequencies (for the frequency format condition) or percentages (for the probability format condition), and the problem was subsequently framed using natural frequencies or percentages respectively. The inputs provided by the participants were adapted to the problem such that the total population was 1,000 events for the frequency format and 100% for the probability format. For instance, if a participant in the frequency format condition stated that the chance of being in the presence of fire in a random school on a random day of the year was 1 out of 5, this was shown on the tree diagram as 200 events where fire occurs, out of 1,000 total events; if he or she stated that the probability of hearing a fire alarm in the case of fire was 9 out of 10, then on the graph the number of events where the fire and alarm occurred were 180 out of the remaining 200 events where fire occurred. In the probability format condition, participants were asked to provide these quantities in the form of percentages and the problem was also framed using percentages. It is worth noting that an inherent property of probability/percentage trees is that the values on the graph are normalized at each branch - i.e., the total number of events is set back to 100% at each node (or to 1 in the case of probabilities). This contrasts with frequency trees in which case the values are derived from a natural sampling process—i.e., each node starts with the number of events which is left from the preceding splits.

The question below the graph asked participants to compute the probability of fire given that the fire alarm was sounding (i.e., the positive predictive value, or PPV). Participants were explicitly asked to calculate the PPV based on the data shown in the graph.

It is worth noting that, in the initial survey, participants were not asked to provide the PPV. This question was asked after the survey, during the experimental task that presented data derived from their responses. Thus, participants could not just rely on memory. They still needed to reason to understand the data, the relationships between different pieces of information and what the question was asking them to calculate.

### 2.2. Study 2

The second study used a different Data Generating Mode. Instead of showing data derived from the participants' personal experience, we displayed fixed values, which were the median of the base rate, true positive rate and false alarm rate values calculated from all the responses given in the first study. As such, they were plausible probabilities, but did not necessarily match people's actual experience with the situation presented in the problem. These values were still collected in the preliminary survey in study 2, in order to calculate the extent to which the difference between participants' reported experience and the average values they were presented with affected performance. Study 2 used a between-subjects design with one factor, Information Format, with two levels (frequency vs. probability).

### 2.3. Participants

The participants were “workers” recruited from Amazon Mechanical Turk (MTurk)^1^, who took part in the study for monetary compensation (Behrend et al., 2011; Mason and Suri, 2012). There were 300 participants in study 1, 150 in each condition, and 300 participants in study 2, 150 in each condition. We eliminated from the analysis those participants who did not disable any active ad-blocker in their web browser (an action which was explicitly requested on the instructions page) before starting the experiment, as the ad-blocker may have interfered with the data collection tool. We also eliminated all those participants who answered the problem without looking at the question at least once. It was possible to detect this from the interaction data, as the participant was required to hover over a button to see the question. Finally, we eliminated from the data set all those participants who did not look at (by hovering over) at least two pieces of information, excluding the question, as this sort of behavior was assumed to indicate a lack of effort from the participants – to answer the question one needs to extract at least two pieces of information from the graph, and this was explicitly mentioned on the problem description page. After eliminating invalid participants based on the above criteria, we were left with 156 participants in study 1 (age range 18–71, 66 males and 90 females) and 186 participants in study 2 (age range 18–68, 65 males and 121 females. The distribution of age and gender of participants across conditions can be found in Table 1. A meta-analysis reviewing 20 years of research on probabilistic reasoning shows that participants with greater educational or professional experience are not better than laypeople at solving probabilistic reasoning problems (McDowell and Jacobs, 2017). However, some research highlights certain links between probabilistic reasoning ability and people's numeracy (Brase and Hill, 2017). Thus, before starting the task, participants were asked to complete the Subjective Numeracy Scale, which is a widely used standardized questionnaire for assessing people's numeracy (Fagerlin et al., 2007). This was used to control for potential confounders stemming from individual differences in mathematical abilities.

**Table 1:** Biographical data and descriptive statistics.

	**Study 1**		**Study 2**	
	**Freq**	**Prob**	**Freq**	**Prob**
Age	35.77(10.54)	37.19(12.76)	34.64(11.14)	34.77(9.78)
Gender	31m/47f	35m/43f	33m/59f	32m/62f
Numeracy	3.88(0.68)	3.98(0.64)	4.05(0.61)	3.67(0.76)
Believed Base Rate	0.05(0.21)	0.10(0.16	0.04(0.24)	0.15(0.21)
Believed True Positive	0.5(0.8)	0.69(0.76)	0.40(0.85)	0.29(0.74)
Believed False Alarm	0.25(0.5)	0.30(0.56)	0.27(0.60)	0.3(0.35)
Estimated PPV	0.22(0.29)	0.45(0.36)	0.22(0.21)	0.37(0.20)
% Correct Estimates	39%	14%	9%	2%

*The values for the descriptive statistics are the means and the standard deviations (in brackets)*.

### 2.4. Procedure and Stimuli

Both studies employed a crowdsourcing method that allocated Amazon Mechanical Turk's Workers to one of the two conditions—frequency format or probability format—counterbalancing the order of the allocation of the participants (Behrend et al., 2011; Mason and Suri, 2012). Those workers who self-enrolled to take part in the study were redirected to our web application, which was hosted on a university server. The application was built in JavaScript and Python and is available on GitHub^2^. The application was specifically designed to display the problem, collect participants' responses, and integrate with another application which was used to track participants' mouse events for the duration of the task (Apaolaza et al., 2013). This is also available on GitHub^3^.

At the beginning of the experiment, an instruction page provided participants with an explanation of the study. Participants were asked to give their consent by checking a box before starting the actual task. After that, demographic data including age and gender were collected, and participants performed the numeracy test. Contextual information was also provided regarding the fire-and-alarm problem, and what was expected from participants (see the Supplementary Materials). Then, participants' estimates of the probability of the three quantities (i.e., base rate, true positive rate, and false alarm rate) were collected. Finally, the actual problem was presented using a tree diagram (see Figure 1), and participants were asked to provide an answer in the dedicated space below the graph, next to the question. After completing the task, participants were redirected to an end-page which provided an alphanumeric code that could be used to retrieve compensation through the Amazon platform.

Inconsistencies between the answers participants gave in the survey and in the actual task could have arisen during the study, due to typographical error, for example. Several checks were thus hard-coded into the web application. For instance, if the numerator was greater than the denominator, the software generated a pop-up window with an error stating that the numerator could not be larger than the denominator.

On the task page, the data were hidden below buttons placed on the tree diagram. The buttons had labels describing the data they concealed (e.g., the button labeled “Fire” covered the number of events with fire). The text describing the question was also hidden behind a button (see Figure 1). To access the concealed data or text, participants had to hover over the relevant button with their mouse. The information was hidden again when they moved away. This interaction technique was used to determine which pieces of information participants thought were relevant, and the order in which they decided to gather these pieces of information.

The advantage of using (explicit) mouse tracking over eye tracking, is that the latter method can include patterns that may not be directly linked to human reasoning, but rather emerge in a bottom up fashion, due, for example, to visual properties of the stimulus (Hornof and Halverson, 2002; Holmqvist et al., 2011; Kok and Jarodzka, 2017). Similarly, continuous mouse movements may not be accurate in determining a user's focus of attention during tasks (Guo and Agichtein, 2010; Huang et al., 2012; Liebling and Dumais, 2014). Studying mouse movements that explicitly uncover information hidden behind buttons means that the events used in the analysis are much closer to conscious cognition.

## 3. Analysis

Two metrics were used to measure participants' performance. The first, Correctness, was a binary variable (correct/incorrect) indicating whether the participant's answer matched the correct answer. For this we applied the extensively used strict rounding criterion proposed by Gigerenzer and Hoffrage, where only those answers matching the true value rounding up or down to the next full percentage point were considered correct answers (Gigerenzer and Hoffrage, 1995).

The second variable, Log-Relative-Error, was a continuous variable measuring how far a participant's answer deviated from the correct answer. This is the result of the function $\log_{10}\left(\frac{Pe}{Pt}\right)$, where Pe is the Estimated Posterior (the given answer) and Pt is the True Posterior, i.e., the answer obtained by applying Bayes' theorem to the data provided on the graph (Micallef et al., 2012; Reani et al., 2018a). Thus, the variable Log-Relative-Error is the log-transformed ratio between the Estimated Posterior and the True Posterior, and indicates an overestimation, if positive, or an underestimation, if negative, of the probability of being in the presence of a fire given that the fire alarm was sounding, with respect to the true probability of such an event. Correct answers result in a value of zero. The full data and the script used for analysis is available on GitHub^4^.

### 3.1. Performance Analysis

In a logistic regression analysis, Correctness served as the response variable, and Information Format, DGM and Numeracy as the predictors. This was fitted to the aggregated data from both studies.

In a linear regression analysis of the data from study 2, Log-Relative-Error served as the response variable and Information Format and Log-Experience-Deviation as the two predictors. Log-Experience-Deviation is the result of the function $\log_{10}\left(\frac{Ps}{Pt}\right)$, where Ps is the Subjective Posterior, i.e., the *a priori* estimate of the risk of fire in the case of an alarm, before seeing the actual data. This value was calculated using the estimates of the base rate, true positive rate and false alarm rate collected during the initial survey. Pt is the True Posterior, as generated using the actual data on the graph.

The value of Log-Experience-Deviation therefore indicates whether a person overestimates, if positive, or underestimates, if negative, the probability of fire in the case of an alarm (i.e., the posterior), in comparison with the real estimate derived using the data presented in the task. A value of zero would result if a participant's estimate based on self-reported lived experience exactly matched the PPV calculated using the aggregated values from study 1.

### 3.2. Mouse Event Analysis

To access an item of information, participants had to hover over the relevant button with a mouse. We designated a meaningful code to each of these locations as defined in Table 2. *T* represents the button covering the total number of events, *F* is the button covering the events with fire, *nF* is the button covering the events with no fire, *FA* is the button covering the events with fire and alarm, *FnA* is the button covering the events with fire and no alarm, *nFA* is the button covering the events with no fire and alarm, *nFnA* is the button covering the events with no fire and no alarm and *Q* is the button covering the question.

**Table 2:** Coding scheme for the locations (i.e., buttons) on the diagram.

**Location Code**	**Description**
T	*Total*
F	*Fire*
nF	*no-Fire*
FA	*Fire and Alarm*
FnA	*Fire and no-Alarm*
nFA	*no-Fire and Alarm*
nFnA	*no-Fire and no-Alarm*
Q	*Question*

Mouse event data was analyzed firstly by considering the proportion of time (as a percentage) spent viewing each location with respect to the total (aggregated) time spent viewing all locations, for each condition. To understand whether there were differences in the order in which people looked at locations between groups, a transition analysis was conducted (Reani et al., 2018a,b). We were interested in determining which locations participants thought were important, and the order in which they accessed these before answering the question. We focused our investigation on bi-grams, calculating for each location the probability that a participant would access each of the other locations next (Reani et al., 2018b, 2019).

The locations thus define a sample space Ω, which includes eight locations in total, Ω = {*T*, *F*, *nF*, *FA*, *FnA*, *nFA*, *nFnA*, *Q*}, from which we derived all possible combinations, without repetition, to form the list of transitions between any two buttons, *L* = 8 × 7 = 56. Once the list of transitions was generated the frequency counts of these were extracted from the interaction data collected for each participant. These values were then normalized by the group total to obtain two frequency distributions of transitions (one for each condition). Then, we calculated the Hellinger distance between these two distributions, as an indicator of the amount of difference in terms of mouse behavior between the frequency format group and the probability format group. A permutation test, which compared the difference between the experimental groups with groups created at random, 10,000 times, was used to determine whether the difference in mouse movement between groups was due to chance, or to the manipulation of the variable of interest (Information Format).

Finally we identified which transitions were the most discriminative, i.e., the transitions that differed most, in term of relative frequency, between the frequency and probability conditions. Two parameters were taken into account to assess whether a transition was a meaningful discriminator. The first is the transition odds-ratio value, calculated as *OR* = $\left(\frac{p}{1-p}\;÷\;\frac{q}{1-q}\right)$ where *p* and *q* are the distributions of transitions in the frequency and probability conditions respectively. The odds-ratio, in this context, is a measure testing the relationship between two variables (Information Format and mouse behavior), and its 95% confidence interval provides a measure of the significance of this relationship. Further details about this method can be found in Reani et al. (2018b, 2019). An odds-ratio of one indicates that the transition is found in both conditions with the same relative frequency, and thus the further from one the odds-ratio is, the more discriminative it is. The second parameter is the maximum frequency *F* = max(*x*_*i*_, *y*_*i*_) - i.e., the maximum value of the transition frequency between the frequency condition and probability condition (Reani et al., 2018b, 2019). A discriminative transition should also have a large *F*, as transitions that occur only a few times are not representative of the strategies used by the majority of people.

We compared participants' mouse behavior between the two formats (frequency vs. probability) in both study 1 and study 2. For study 1 only, we also compared the mouse behavior of correct and incorrect respondents, for both conditions (frequency and probability format) separately, to determine whether participants who answered correctly exhibited different mouse behavior from participants who answered incorrectly. This is because the number of correct responses was large enough to support a meaningful comparison only in study 1. In this latter analysis, the odds-ratio scale is a measure of the relationship between Correctness and mouse behavior.

## 4. Results

The results are reported separately for each study and for each condition (probability vs. frequency), for the variables Correctness and Log-Relative-Error. When reporting results for the variable Numeracy, we aggregated the data of both studies. The results for the variable Log-Experience-Deviation are reported for study 2 only.

### 4.1. Performance Analysis Results

In the experience-matched data mode (study 1), 39% of the participants presented with the frequency format answered correctly, but only 14% of those presented with the probability format. In the experience-mismatched data mode (study 2), 9% of the participants answered correctly with the frequency format, and only 2% with the probability format. Thus more people answered correctly with the frequency format, regardless of the Data Generating Mode, and more people answered correctly with the experience-matched data mode, regardless of the Information Format.

The descriptive statistics for the variable Numeracy are reported for Correctness and Information Format separately, aggregating the data from both studies. For incorrect respondents in the frequency condition, the Numeracy median was *Mdn* = 3.88 (IQR = 0.88), for incorrect respondents in the probability condition, *Mdn* = 3.89 (IQR = 1.01), for correct respondents in the frequency condition, *Mdn* = 4.19 (IQR = 0.69), and for correct respondents in the probability condition, *Mdn* = 4.17 (IQR = 0.75). From these results, it appears that correct respondents were, on average, slightly more numerate than incorrect respondents. The full descriptive statistics are reported in Table 1.

A logistic regression analysis, with Correctness as the response variable and Information Format, DGM and Numeracy as predictors shows that Information Format was a strong predictor of Correctness (odds ratio OR = 0.23, 95% Confidence Interval CI [0.12, 0.47]), indicating that the odds of answering correctly in the frequency format were four times the odds of answering correctly in the probability format. The logistic model shows that Data Generating Mode was also a strong predictor of Correctness (OR = 0.14, 95% CI [0.07, 0.29]), indicating that the odds of answering correctly in the experience-matched data mode were about 7 times the odds of answering correctly in the experience-mismatched data mode. Numeracy was not a strong predictor of Correctness (OR = 1.65, 95% CI [0.82, 3.32]).

As reported by Weber and colleagues, performance in Bayesian reasoning tasks seems to improve when no false negatives are present in the problem description; i.e., when the hit rate is 100% (Weber et al., 2018). If some of the participants, in study 1, were presented with a problem with no false negatives, this could potentially have influenced the results of the regression analysis. In study 1, there were only seven participants who were presented with a problem with a hit rate of 100%, and another two who were presented with a problem with a hit rate higher than 99%. To exclude potential confounders stemming from problems with a hit rate equal or close to 100%, we re-ran the analysis excluding these participants from the dataset. This did not significantly change the results (see Supplementary Materials).

As the number of correct responses was limited, four additional 2x2 chi-squared tests were performed to assess whether there was a real relationship between Information Format and Correctness, one for each study, and between DGM and Correctness, one for each Information Format (the p-values reported below are adjusted using the Bonferroni method for multiple comparisons). The first chi-squared test of independence revealed that, in study 1, Information Format was significantly associated with Correctness, χ^2^(1, N = 156) = 11.762, *p* = 0.002). Cramer's *V* determined that these variables shared 28% variance. The second chi-squared test of independence revealed that, in study 2, Information Format was marginally associated with Correctness, χ^2^(1, N = 186) = 3.617, *p* = 0.057). Cramer's *V* determined that these variables shared 14% variance. The third chi-squared test of independence revealed that, in study 2, DGM was significantly associated with Correctness, χ^2^(1, N = 170) = 19.427, *p* < 0.001). Cramer's *V* determined that these variables shared 33% variance. The fourth chi-squared test of independence revealed that, in study 2, DGM was also significantly associated with Correctness, χ^2^(1, N = 172) = 7.11, *p* = 0.03). Cramer's *V* determined that these variables shared 20% variance.

The medians for the variable Log-Relative-Error were *Mdn* = 0.01 (IQR = 0.33) for the frequency format in study 1, *Mdn* = 0.30 (IQR = 0.68) for the probability format in study 1, *Mdn* = -0.23 (IQR = 0.90) for the frequency format in study 2 and *Mdn* = 0.46 (IQR = 0.53) for the probability format in study 2. It can be noted that the relative error was considerably larger for the probability format than the frequency format, and relatively larger in study 2 compared with study 1.

**Figure 2:**
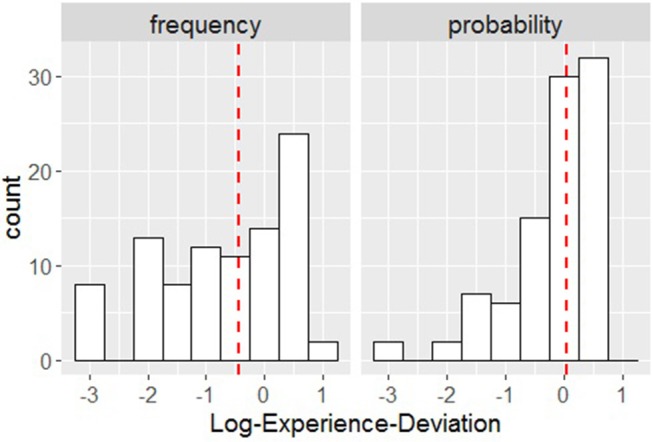
Distribution of Log-Experience-Deviation for frequency **(Left)** and probability **(Right)**; the vertical red dashed lines represent the medians.

The medians for the variable Log-Experience-Deviation (in study 2 only) were *Mdn* = -0.42 (IQR = 1.92) for the frequency format and *Mdn* = 0.02 (IQR = 0.86) for the probability format. On average, the Subjective Posterior was considerably closer to the True Posterior for the probability format compared with the frequency format. Participants using the frequency format estimated that, on average, the probability of fire in the case of hearing a fire alarm was considerably smaller (*Mdn* = 0.07) than the probability presented in the task (*Mdn* = 0.17; this latter median is the value derived from the data collected in study 1; the other median values from study 1 were base rate = 0.1, true positive rate = 0.5, false alarm rate = 0.27). The median of the answers provided for the probability format was *Mdn* = 0.18, which is very close to the true value of 0.17. This indicates that, in study 2, participants' estimates in the probability format condition were similar to the estimates that participants in study 1 made about the risk of fire (see Figure 2).

In the second (linear) regression, conducted for study 2 only, we used Log-Relative-Error as the response variable and Information Format and Log-Experience-Deviation as the predictors. The second regression model was fitted to the data from study 2 only, as in study 1 the data presented for calculating the correct answers were derived from participant's reported experience (collected in the initial survey). In study 1 Log-Experience-Deviation is therefore a constant with a value of zero. The results from the regression indicate a significant effect of both predictors on the response variable Log-Relative-Error. For Information Format (with frequency format as the reference class), Beta = 0.26, 95% CI [0.13, 0.39]) and for Log-Experience-Deviation, Beta = 0.10, 95% CI [0.04, 0.16]. Thus, the probability format was associated with a 1.30 increase in the relative error, compared with the frequency format. This indicates that the use of probabilities produced a larger deviation in participants' estimates. The analysis also shows that with a one unit increase in the deviation of the Subjective Posterior from the True Posterior, the relative error in the estimate increased by 0.41 units, on average.

This result suggests that the more participants' self-reported lived experience differed from the actual data presented, the larger their over- or underestimate in the direction of those beliefs; the larger the deviation of the (*a priori*) Subjective Posterior from the True Posterior, the larger the deviation of the (*a posteriori*) Estimated Posterior from the True Posterior. This result suggests that the larger the deviation of the (a priori) Subjective Posterior (derived from participants' self-reported lived experience) from the True Posterior (derived from the problem data), the larger the deviation of the (a posteriori) Estimated Posterior (participants' answer) from the True Posterior. The bias in participants' response was also in the direction of their beliefs. This indicates that there is a tendency for people to give an answer consistent with their personal experience rather than the data provided.

### 4.2. Interaction Analysis Results

The interaction analysis is divided in two parts. The first part focuses on analyzing the amount of time participants spent on different locations of interest, comparing the two conditions (frequency vs. probability format). The second part focuses on analyzing the order in which these locations are visited by participants, looking for repetitive patterns within groups.

#### 4.2.1. Dwell Time

The variable Dwell Time, measured as a percentage, is the amount of time viewing a location (hovering over a button) on the graph divided by the total time spent viewing all locations. This is reported in Table 3, by condition (frequency vs. probability) and by study (study 1 vs. study 2). The table also reports *d* which is the difference between the mean Dwell Time for the frequency format and the mean Dwell Time for the probability format, divided by the pooled standard deviation. Here we only report the two largest *d* values in both studies. For the full results see Table 3.

**Table 3:** Means (*M*) and standard deviations (*SD*), for Dwell Time in percentages for each location, for the frequency format (left) and probability format (right), and for study 1 (top) and study 2 (bottom).

**Location**	**Freq *M***	**Freq *SD***	**Prob *M***	**Prob *SD***	***d***
**Study 1**					
T	9	7	7	7	0.29
F	11	9	10	9	0.11
nF	11	11	11	10	0.00
FA	9	7	12	9	0.37
FnA	5	4	7	6	0.39
nFA	7	6	8	10	0.12
nFnA	7	7	9	10	0.23
Q	42	17	36	16	0.36
**Study 2**					
T	12	8	7	7	0.67
F	9	7	11	8	0.27
nF	10	9	11	8	0.12
FA	10	9	10	7	0.00
FnA	5	7	7	7	0.29
nFA	7	7	6	8	0.13
nFnA	7	9	8	7	0.12
Q	39	17	41	16	0.12

*The table also reports the standardized difference in means by condition (d)*.

The largest relative difference in study 1 was found in location FnA (fire and no alarm), with participants in the probability condition (*M* = 7%, *SD* = 6) spending a larger proportion of time, on average, viewing this location than participants in the frequency condition (*M* = 5%, *SD* = 4). The second largest relative difference in study 1 was found in location FA (fire and alarm), where participants in the probability condition (*M* = 12%, *SD* = 9) spent a larger proportion of time, on average, than participants in the frequency condition (*M* = 9%, *SD* = 7).

The largest relative difference in study 2 was found in location T (the total number of events), where participants in the frequency condition (*M* = 12%, *SD* = 8) spent a larger proportion of time, on average, than participants in the probability condition (*M* = 7%, *SD* = 7). The second largest relative difference in study 2 was found in location FnA (fire and no alarm), where participants in the probability condition (*M* = 7%, *SD* = 7) spent a larger proportion of time, on average, than participants in the frequency condition (*M* = 5%, *SD* = 7).

A consistent pattern found in both studies was that participants presented with the frequency format tended to spend more time on location T, and participants presented with the probability format tended to spend more time on location FnA. Moreover, in both studies, participants in the probability format condition tended to focus more on the upper branch of the Tree, represented by locations F, FA and FnA, compared with participants using the frequency format (see Table 3).

#### 4.2.2. Permutation Tests

For study 1 and study 2, separate permutation tests, with 10,000 permutations each, compared the Hellinger distance between the distribution of transitions for the frequency format and the distribution of transitions for the probability format, with the distance between two distributions created at random (Reani et al., 2018b).

**Figure 3:**
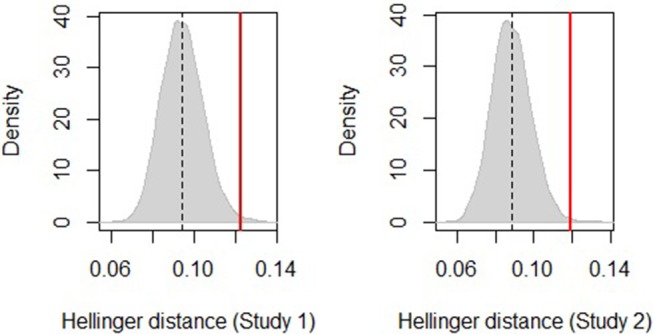
Sampling distribution of distances between the frequency and the probability groups for study 1 the left and study 2 on the right. The vertical red line is the actual Hellinger distance between groups.

The estimated sampling distributions of the two tests are shown in Figure 3. The vertical red line represents the distance between the frequency and the probability groups for study 1, on the left, and for study 2, on the right. The gray curve represents the distributions of the distances between pairs of randomly sampled groups (with replacement) of comparable sizes. The cut-off area under the curve delimited to the right of the vertical line is the probability of the null hypothesis being true; i.e., that the distance between the transition distributions of the frequency and the probability groups is not different from the distances between any two groups of comparable sizes sampled at random from the population.

The permutation test for study 1 shows a significant difference between the frequency and the probability conditions: the Hellinger distance is *Hd* = 0.123 and the p-value is *p* = 0.005. A similar effect was found for study 2 (*Hd* = 0.119, *p* = 0.002). These results indicate that participants' mouse behavior differed between Information Format groups, in both studies.

For study 1 only, we ran two further permutation tests to investigate whether Correctness was also related to participants' mouse behavior in the frequency and probability conditions respectively. The comparison between transitions for the correct respondents and transitions for the incorrect respondents is meaningful only if there are enough participants who answered the problem correctly (Reani et al., 2018b, 2019). Thus, we did not run these tests on the participants who took part in the second study because in study 2 the number of correct responses was too small to enable a meaningful comparison. The results for the frequency condition did not show a significant difference between Correct and Incorrect groups – the Hellinger distance was *Hd* = 0.11 and the p-value was *p* = 0.21. A similar result was found for the probability condition (*Hd* = 0.17, *p* = 0.80). These results indicate that participants' mouse behavior was not related to the variable Correctness.

#### 4.2.3. Discriminative Transitions

The results from the first set of permutation tests suggest that, in both studies, there were mouse transitions that might typify users' behavior in different Information Format conditions.

**Figure 4:**
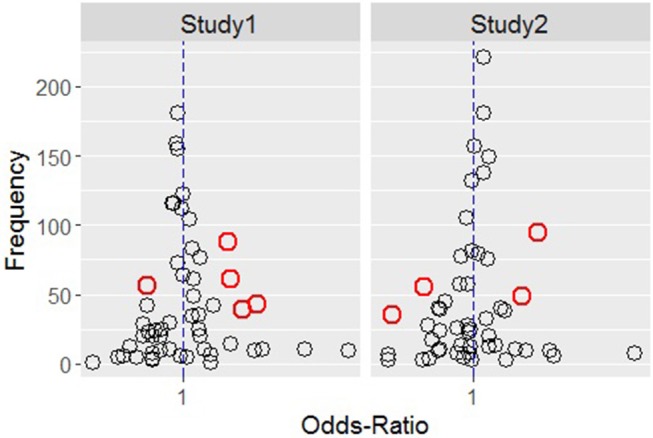
Transitions distribution by odds-ratio (x-axis) and absolute frequency (y-axis) for study 1 **(Left)** and study 2 **(Right)** conditions.

Figure 4 shows, for study 1 on the left and for study 2 on the right, all the transitions by OR on the *x*-axis (scaled using a logarithmic transformation) and by absolute frequency on the *y*-axis.

The red circles are those transitions that have a narrow confidence interval that does not include the value one. These tend to be the transitions which have an *OR* far from one (represented in the graph by the vertical dashed blue line) and, at the same time, a relatively large *F*. Table 4 reports these transition together with their *OR* values, confidence intervals and frequency, for study 1 (top) and study 2 (bottom).

**Table 4:** Discriminative Transitions by Study, with odds-ratio values, 95% confidence intervals and absolute frequency of occurrence.

**Study**	**Trans**	**OR**	**95% CI**	**Freq**
1	F-T	1.69	1.15–2.49	88
1	nF-T	1.99	1.09–3.68	39
1	FnA-F	2.37	1.28–4.41	43
1	nFA-nF	1.74	1.09–2.77	61
1	Q-T	0.65	0.44–0.94	56
2	F-T	2.13	1.44–3.17	95
2	nF-T	1.78	1.07–2.97	49
2	FnA-nF	0.56	0.37–0.84	55
2	nF-Q	0.39	0.22–0.67	35

In Table 4, an *OR* value larger than one indicates a larger relative frequency for that transition in the frequency format compared with the probability format. There were five discriminative transitions in study 1, four of which represented the typical behavior of participants presented with the frequency format (F-T, nF-T, FnA-F and nFA-nF) and one which represented the typical behavior of participants presented with the probability format (Q-T). In study 2, we found four discriminative transitions, two of which represented the typical behavior of participants presented with the frequency format (F-T and nF-T), and two which represented the typical behavior of participants presented with the probability format (FnA-nF and nF-Q).

**Figure 5:**
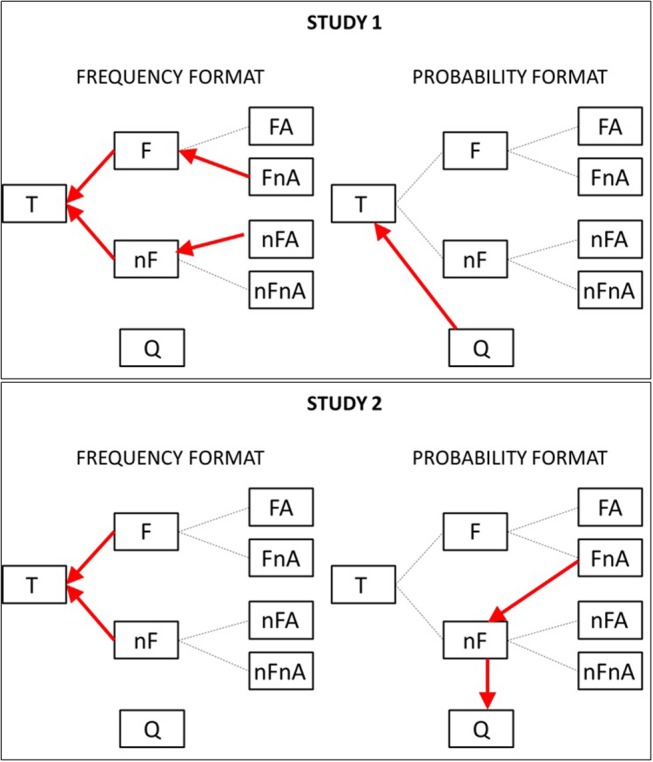
Discriminative transitions shown using arrows on the original tree diagram, for the frequency **(Left)** and probability **(Right)** conditions, and for study 1 **(Top)** and study 2 **(Bottom)**.

To understand what these transitions represent in the context of the problem, we mapped them onto the original tree diagram in Figure 5, where red arrows represent the discriminative transitions for the frequency format (right) and the probability format (left) and for study 1 (top) and study 2 (bottom).

From the graph, it can be noted that, in study 1, participants in the frequency condition were more likely than participants in the probability condition to move leftwards, toward the total number of events (location T). In the probability format, they tended to move upwards, from location Q (the question) to location T (the total number of events).

In study 2, the pattern found in study 1 is repeated, i.e., participants in the frequency format tended to move leftwards, from the events with fire to the total number of events (F-T) and from the event with no-fire to the total (nF-T). Participants in the probability condition tended to move downwards, from the location representing the events with fire and no-alarm to the events with no-fire (FnA-nF) and from this latter location to the question (location Q).

## 5. Discussion

This research investigated the effects of Information Format (whether data is presented in frequencies or probabilities) and Data Generating Mode (whether or not the data directly matched an individual's self-reported lived experience), on how people approach probabilistic reasoning tasks (Gigerenzer, 2015; Hoffrage et al., 2015). To determine whether there were differences in reasoning behavior between conditions, it employed a novel interaction analysis approach in an online task. In line with previous research, we found that people were more likely to provide an accurate answer when presented with data in the frequency format than the probability format (Gigerenzer and Hoffrage, 1995; Gigerenzer, 2015; McDowell and Jacobs, 2017). In support of our first hypothesis (H1), we found that people were more likely to answer accurately when presented with data that matched their reported experience, than when they were presented with data that matched the average person's experience, and that the extent to which their answer deviated from the correct response in study 2 was directly related to the distance between the subjective posterior and the true posterior. This provides support for the idea that experiencing data is strongly related to being able to reason about it correctly (Gigerenzer, 2015; Sirota et al., 2015; Hertwig et al., 2018). It also demonstrates that the effect of this learned subjective posterior (here, the result of lived experience) may hinder people's ability to reason about information that does not match it.

The results did not support our second hypothesis (H2) as the manipulation of the format *did* have an effect regardless of DGM. This suggests that the difference in performance found in previous research comparing the frequency and probability formats is not due solely to the former being able to trigger the perception of learning from experience, but rather that, in line with the nested-set hypothesis, the facilitatory effect of the frequency format is due to a clearer representation of the relationships between sets (Sirota and Juanchich, 2011; Lesage et al., 2013; Sirota et al., 2015). We tested our third hypothesis (H3) – that mouse movement would differ according to the format in which the information is encoded – by using a web-based tool that forced people to hover the mouse cursor over those parts of the graph that the participants thought were crucial for solving the problem, and analyzing the differences in transitions between these locations.

In both studies, participants using the frequency format tended to focus more on the total number of events (location T), compared with participants using the probability format. It was also the case in both studies that participants in the probability format condition tended to focus more on location FnA (fire and no alarm) than participants in the frequency format condition. The question asks participants to estimate the probability of fire, given that the alarm sounded. Thus, looking at FnA is not necessary to answer the question. The only useful locations for solving the problem framed using probabilities are FA, nFA, F and nF, which were the pieces of data that had to be entered in the Bayesian formula to produce the correct estimate. One possible reason why people looked more at location FnA in the probability format condition, might be that the normalization process used in the probability Tree is not clear, and thus people look at the opposite data value in an attempt to understand how the data were normalized.

**Figure 6:**
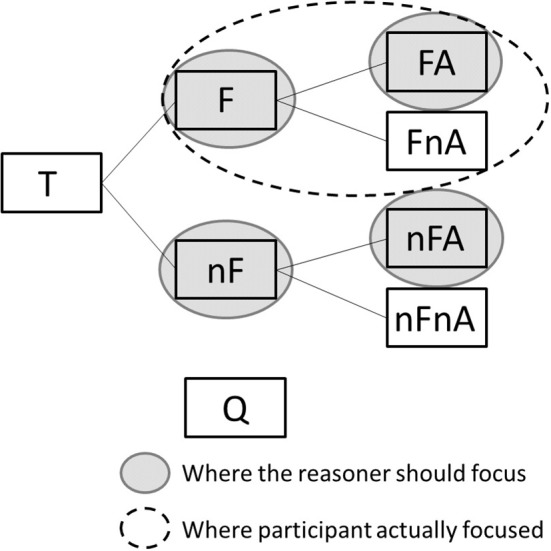
The probability format condition, marked to show where the reasoner should focus (gray-filled ellipses) and where participants actually focused (dashed-line ellipses).

A second explanation is that people focus on this because they are trying to compare events with alarm and events without alarm given that there was a fire, confusing the sensitivity of the test with the PPV. This may explain why participants in the probability format tended to focus more on the information found on the upper branch of the tree, which shows only the data related to events with fire (see Table 3). This interpretation is represented in Figure 6 which shows, for the probability format condition, where the reasoner should focus to answer the question correctly (gray-filled circles), and where participants actually focused in the experiment (dashed-line circles).

The answer analysis showed that in the probability format condition 60% of participants in study 1 and 52% of participants in study 2 gave the value of the sensitivity of the test, instead of the PPV, as the answer to the problem. This reflects the mouse movement patterns described above (related to the second explanation). This result is also in line with previous research showing that the error made most often by participants, in probabilistic reasoning tasks framed using probabilities, is confusing the sensitivity of the tests with the PPV (Eddy, 1982; Elstein, 1988; Gigerenzer et al., 1998; Hoffrage and Gigerenzer, 1998). From our results, it appears that mouse behavior does indeed provide evidence for this faulty reasoning strategy. The transition analysis shows that participants, in the frequency format only, have the tendency to move the cursor leftwards, toward the total number of events (T). This ‘reversion to total’ behavior, which is found in both studies, is also found in research using eye tracking methods to study a similar problem (Reani et al., 2019). These results also reflect the responses provided by many of the participants. When presented with the frequency format, 45% of the participants in study 1 and 57% of the participants in study 2 used, as the denominator of the proportion in their answer, the total number of events (i.e., 1,000). This value was covered by the button T, and these results might explain why a great number of participants exhibit the behavior of going leftwards often, toward T. This provides behavioral evidence that, as proposed in other studies, the most common error of participants in the frequency format is to use the total population, instead of the relevant subset (i.e, alarm events) as the denominator in the answer, perhaps because they did not understand which population the question refers to (Khan et al., 2015; Reani et al., 2018a, 2019). When presented with the probability format, participants tended to move vertically, from the question toward location T (study 1) and from location nF toward the question (study 2). This suggests that participants tend to check the question more often when the problem is framed using probabilities, perhaps because the question in this case is more difficult to understand compared with when the problem is framed using frequencies. This confusion is in line with the fact that a significantly larger number of participants answered incorrectly when the problem was presented using probabilities. Although we found interesting correlates between participants' mouse behavior and their answers, this method has two main limitations. The first is that *post hoc* analyses of this type leave room for different interpretations. Here we interpret our results in terms of current theory and participants' responses.

The second limitation is that the experimental settings used in the current study were different from the settings used in previous research. Specifically, in our studies, the data were not available to the participants all of the time; participants needed to move the mouse to see the values hidden behind buttons, and this has the potential to change the reasoning process. In tasks where the data is always available, people have immediate access to the information and some aspects of this information may be taken on board implicitly and effortlessly. In tasks where the information is covered and people need to engage interactively with the tool to uncover the data, certain implicit processes that should occur in the data acquisition stage may be lost. This loss, however, can be beneficial for the purpose of studying conscious human cognition, especially in tasks involving complex reasoning, because it filters out some of those noisy patterns that are associated with low-level perceptions.

It is assumed that the fire-and-alarm scenario used in this study is a familiar situation to most of the participants (at least compared to the mammography problem). Nevertheless, we cannot be sure that the participants were all familiar with such a scenario. As a consequence of using a single scenario, we cannot be sure that these results would generalize to other familiar/everyday scenarios as well.

Throughout the study we kept the settings of the problem constant and we provided the same information in all the conditions. We manipulated only the format (probability vs. frequency) and whether the data provided matched people's reported experience. It is possible other factors may have affected the results. For instance, some participants may have been more familiar with the scenario compare to others, perhaps because they were firefighters.

Previous research has explored eye-mouse coordination patterns, for instance, in tasks where participants were asked to examine SERP (Guo and Agichtein, 2010). However, such a comparison has not been conducted in probabilistic reasoning research. Thus, future work will focus on combining different interaction analysis methods such as eye tracking and mouse tracking simultaneously, to understand what each can tell us about the reasoning process.

Although the study was not a memory test, as the data were available for the whole duration of the task, it is possible that, in study 1, familiarity with the uncertainty surrounding the event may have potentially lessened the load in working memory while performing calculations. To exclude any memory effect, this needs to be further investigated in further.

There is evidence that the tendency to use the total sample size as the denominator of the Bayesian ratio has been related to a superficial processing of the data (Tubau et al., 2018). The sequential presentation of isolated numbers might thus be linked to a more superficial processing. Future work could investigate this by comparing the effect of presenting complete uncovered trees vs. presenting covered trees, of the type used in this study.

A further limitation of the study is the use of the word “events” in the question, when referring to days in which there was a fire. Although the problem description uses “days” to describe the scenario, the question then asks participants to provide the number of events, which is an ambiguous term. Some participants may have had misunderstood what this term referred to.

Relatedly, the fact that the mean of the base rate across conditions ranges from 4 to 15% (i.e., it is relatively high), might indicate that some participants did not have a good “feeling” for the real base rate related to the scenario. This might need to be explored in future work as the scenario was chosen to be a familiar one when in fact, for some participants, this might not have been the case.

## 6. Conclusion

We investigated how Information Format affected mouse behavior in an interactive probabilistic reasoning task, and whether presenting probabilities that matched people's self-reported lived experience improved the accuracy of their posterior probability estimates. We found that the closer the data presented in the task were to self-reported experience, the more accurate people's answers were, indicating that the subjective posterior developed through lived experience had an overwhelming impact on the reasoning process. We also found that people are better able to reason about data presented in frequencies regardless of whether they match experience. By analyzing mouse events in light of participants' responses, we obtained evidence for different faulty strategies related to frequency presentation and probability presentation respectively. This supports analysis of mouse behavior as a way of gathering evidence about the cognitive processes underpinning probabilistic reasoning.

## Data Availability

Publicly available datasets were analyzed in this study. This data can be found here: https://github.com/manurea/mouse-interaction-bayesian-reasoning.

## Ethics Statement

This study was carried out in accordance with the recommendations of the Computer Science School Panel at The University of Manchester with written informed consent from all subjects. All subjects gave written informed consent in accordance with the Declaration of Helsinki. The protocol was approved by The University of Manchester ethics committee.

## Author Contributions

MR wrote the manuscript, designed the experiments, collected the data, and performed the analysis. AD helped with software development. NP supervised the project and advised on the statistical analysis. CJ supervised the project and edited the manuscript.

### Conflict of Interest Statement

The authors declare that the research was conducted in the absence of any commercial or financial relationships that could be construed as a potential conflict of interest.

## Footnotes

^1^Amazon Mechanical Turk: https://www.mturk.com/

^2^GitHub: https://github.com/IAM-lab/FireWeb

^3^GitHub: https://github.com/aapaolaza/UCIVIT-WebIntCap

^4^GitHub: https://github.com/manurea/study4

## References

[B1] Apaolaza A. Harper S. Jay C. (2013). Understanding users in the wild, in W4A '13 Proceedings of the 10th International Cross-Disciplinary Conference on Web Accessibility (New York, NY: ACM). 10.1145/2461121.2461133

[B2] Bar-Hillel M. (1983). The base rate fallacy controversy, in Decision Making Under Uncertainty, Advances in Psychology, vol 16, ed Scholz R. W. 39–61. 10.1016/S0166-4115(08)62193-7

[B3] Behrend T. S. Sharek D. J. Meade A. W. Wiebe E. N. (2011). The viability of crowdsourcing for survey research. Behav. Res. Methods 43:800. 10.3758/s13428-011-0081-0 21437749

[B4] Binder K. Krauss S. Bruckmaier G. (2015). Effects of visualizing statistical information–an empirical study on tree diagrams and 2 ×2 tables. Front. Psychol. 6:1186 10.3389/fpsyg.2015.01186 26379569 PMC4549558

[B5] Binder K. Krauss S. Bruckmaier G. Marienhagen J. (2018). Visualizing the bayesian 2-test case: the effect of tree diagrams on medical decision making. PLoS ONE 13:e0195029. 10.1371/journal.pone.0195029 29584770 PMC5871005

[B6] Böcherer-Linder K. Eichler A. (2017). The impact of visualizing nested sets. an empirical study on tree diagrams and unit squares. Front. Psychol. 7:2026. 10.3389/fpsyg.2016.02026 28123371 PMC5226638

[B7] Brase G. L. Hill W. T. (2017). Adding up to good bayesian reasoning: problem format manipulations and individual skill differences. J. Exp. Psychol. General 146:577–591. 10.1037/xge0000280 28383994

[B8] Cohen A. L. Sidlowski S. Staub A. (2017). Beliefs and bayesian reasoning. Psychon. Bull. Rev. 24, 972–978. 10.3758/s13423-016-1161-z 27604495

[B9] Cosmides L. Tooby J. (1996). Are humans good intuitive statisticians after all? rethinking some conclusions from the literature on judgment under uncertainty. Cognition 58, 1–73.

[B10] Cosmides L. Tooby J. (2008). Can a general deontic logic capture the facts of human moral reasoning? how the mind interprets social exchange rules and detects cheaters, in Moral Psychology, ed Sinnott-Armstrong (Cambridge, MA: MIT Press), W53–W119.

[B11] Eddy D. M. (1982). Probabilistic reasoning in clinical medicine: Problems and opportunities, in Judgment Under Uncertainty: Heuristics and Biases, eds Kahneman D. Slovic P. Tversky A. (Cambridge: Cambridge University Press), 249–267.

[B12] Edwards W. (1968). Conservatism in human information processing, Formal Representation of Human Judgment, ed Kleinmuntz B. (New York, NY: John Wiley & Sons Inc).

[B13] Edwards W. (1982). Conservatism in Human Information Processing. Cambridge: Cambridge University Press, 359–369.

[B14] Elstein A. S. (1988). Cognitive processes in clinical inference and decision making, in Reasoning, Inference, and Judgment in Clinical Psychology ed Salovey D. C. T. P. (New York, NY: Free Press), 17–50.

[B15] Fagerlin A. Zikmund-Fisher B. J. Ubel P. A. Jankovic A. Derry H. A. Smith D. M. (2007). Measuring numeracy without a math test: development of the Subjective Numeracy Scale. Med. Decision Making 27, 672–680. 10.1177/0272989X07304449 17641137

[B16] Gigerenzer G. (2015). On the supposed evidence for libertarian paternalism. Rev. Philos. Psychol. 6„ 361–383. 10.1007/s13164-015-0248-1 26213590 PMC4512281

[B17] Gigerenzer G. Hoffrage U. (1995). How to improve bayesian reasoning without instruction: frequency formats. Psychol. Rev. 102:684.

[B18] Gigerenzer G. Hoffrage U. (2007). The role of representation in bayesian reasoning: correcting common misconceptions. Behav. Brain Sci. 30, 264–267. 10.1017/S0140525X07001756

[B19] Gigerenzer G. Hoffrage U. Ebert A. (1998). Aids counselling for low-risk clients. AIDS Care 10, 197–211. 9625903 10.1080/09540129850124451

[B20] Guo Q. Agichtein E. (2010). Ready to buy or just browsing?: Detecting web searcher goals from interaction data, in SIGIR '10 Proceedings of the 33rd International ACM SIGIR Conference on Research and Development in Information Retrieval (New York, NY: ACM), 130–137.

[B21] Hertwig R. Barron G. Weber E. U. Erev I. (2004). Decisions from experience and the effect of rare events in risky choice. Psychol. Sci. 15, 534–539. 10.1111/j.0956-7976.2004.00715.x 15270998

[B22] Hertwig R. Hogarth R. M. Lejarraga T. (2018). Experience and description: exploring two paths to knowledge. Curr. Direct. Psychol. Sci. 27, 123–128. 10.1177/0963721417740645

[B23] Hoffrage U. Gigerenzer G. (1998). Using natural frequencies to improve diagnostic inferences. Acad. Med. 73, 538–540. 10.1097/00001888-199805000-00024 9609869

[B24] Hoffrage U. Gigerenzer G. Krauss S. Martignon L. (2002). Representation facilitates reasoning: What natural frequencies are and what they are not. Cognition 84, 343–352. 10.1016/S0010-0277(02)00050-1 12044739

[B25] Hoffrage U. Krauss S. Martignon L. Gigerenzer G. (2015). Natural frequencies improve bayesian reasoning in simple and complex inference tasks. Front. Psychol. 6:1473. 10.3389/fpsyg.2015.01473 26528197 PMC4604268

[B26] Holmqvist K. Nyström M. Andersson R. Dewhurst R. Jarodzka H. Van de Weijer J. (2011). Eye Tracking: A Comprehensive Guide to Methods and Measures. Oxford: Oxford University Press.

[B27] Hornof A. J. Halverson T. (2002). Cleaning up systematic error in eye-tracking data by using required fixation locations. Behav. Res. Methods Instr. Comput. 34, 592–604. 10.3758/BF03195487 12564562

[B28] Huang J. White R. Buscher G. (2012). User see, user point: gaze and cursor alignment in web search, in Proceedings of the SIGCHI Conference on Human Factors in Computing Systems (New York, NY: ACM), 1341–1350.

[B29] Khan A. Breslav S. Glueck M. Hornbæk K. (2015). Benefits of visualization in the Mammography Problem. Int. J. Hum. Comput. Stud. 83, 94–113. 10.1016/j.ijhcs.2015.07.001

[B30] Kok E. M. Jarodzka H. (2017). Before your very eyes: the value and limitations of eye tracking in medical education. Med. Educ. 51, 114–122. 10.1111/medu.13066 27580633

[B31] Lesage E. Navarrete G. De Neys W. (2013). Evolutionary modules and bayesian facilitation: the role of general cognitive resources. Think. Reas. 19, 27–53. 10.1080/13546783.2012.713177

[B32] Liebling D. J. Dumais S. T. (2014). Gaze and mouse coordination in everyday work, in Proceedings of the 2014 ACM International Joint Conference on Pervasive and Ubiquitous Computing: Adjunct Publication (New York, NY: ACM), 1141–1150.

[B33] Mandel D. R. (2014). The psychology of bayesian reasoning. Front. Psychol. 5:1144. 10.3389/fpsyg.2014.01144 25346713 PMC4191302

[B34] Mason W. Suri S. (2012). Conducting behavioral research on amazon's mechanical turk. Behav. Res. Methods 44, 1–23. 10.3758/s13428-011-0124-6 21717266

[B35] Mayer R. (2005). Cognitive theory of multimedia learning, in The Cambridge Handbook of Multimedia Learning eds Fagerberg J. Mowery D. C. Nelson R. R. (Cambridge: Cambridge University Press), 31–48.

[B36] McDowell M. Jacobs P. (2017). Meta-analysis of the effect of natural frequencies on bayesian reasoning. Psychol. Bull. 143:1273–1312. 10.1037/bul0000126 29048176

[B37] Micallef L. Dragicevic P. Fekete J. (2012). Assessing the effect of visualizations on bayesian reasoning through crowdsourcing. IEEE Trans. Visual. Comput. Graph. 18, 2536–2545. 10.1109/TVCG.2012.199 26357162

[B38] Ottley A. Peck E. M. Harrison L. T. Afergan D. Ziemkiewicz C. Taylor H. A.. (2016). Improving bayesian reasoning: The effects of phrasing, visualization, and spatial ability. IEEE Trans. Visual. Comput. Graph. 22, 529–538. 10.1109/TVCG.2015.2467758 26390491

[B39] Phillips L. D. Edwards W. (1966). Conservatism in a simple probability inference task. J. Exp. Psychol. 72:346. 10.1037/h0023653 5968681

[B40] Reani M. Davies A. Peek N. Jay C. (2018a). How do people use information presentation to make decisions in bayesian reasoning tasks? Int. J. Hum. Comput. Stud. 111, 62–77. 10.1016/j.ijhcs.2017.11.004

[B41] Reani M. Peek N. Jay C. (2018b). An investigation of the effects of n-gram length in scanpath analysis for eye-tracking research, in Proceedings of the 2018 ACM Symposium on Eye Tracking Research & Applications (New York, NY: ACM). 10.1145/3204493.3204527

[B42] Reani M. Peek N. Jay C. (2019). How different visualizations affect human reasoning about uncertainty: an analysis of visual behaviour. Comput. Hum. Behav. 92, 55–64. 10.1016/j.chb.2018.10.033

[B43] Schulte-Mecklenbeck M. Kuehberger A. Johnson J. G. (2019). A Handbook of Process Tracing Methods, 2nd Ed. New York, NY: Routledge.

[B44] Sirota M. Juanchich M. (2011). Role of numeracy and cognitive reflection in bayesian reasoning with natural frequencies. Studia Psychol. 53, 151–161. Available online at: https://www.scopus.com/record/display.uri?eid=2-s2.0-79960000436&origin=inward&txGid=20f0bffecbf43b0d39147c29faea8c5c

[B45] Sirota M. Vallée-Tourangeau G. Vallée-Tourangeau F. Juanchich M. (2015). On bayesian problem-solving: helping bayesians solve simple bayesian word problems. Front. Psychol. 6:1141. 10.3389/fpsyg.2015.01141 26321977 PMC4530256

[B46] Slovic P. Lichtenstein S. (1971). Comparison of bayesian and regression approaches to the study of information processing in judgment. Organ. Behav. Hum. Perform. 6, 649–744.

[B47] Sweller J. (2003). Evolution of human cognitive architecture, in Psychology of Learning and Motivation, Vol 43 (Academic Press), 215–266. Available online at: https://www.sciencedirect.com/science/article/pii/S0079742103010156

[B48] Traczyk J. Sobkow A. Matukiewicz A. Petrova D. Garcia-Retamero R. (2019). The experience-based format of probability improves probability estimates: The moderating role of individual differences in numeracy. Int. J. Psychol. [Epub ahead of print]. 10.1002/ijop.12566 30690731

[B49] Tubau E. Rodríguez-Ferreiro J. Barberia I. Colomé À. (2018). From reading numbers to seeing ratios: a benefit of icons for risk comprehension. Psychol. Res. [Epub ahead of print].1–9. 10.1007/s00426-018-1041-4 29931591

[B50] Tversky A. Kahneman D. (1974). Judgment under uncertainty: heuristics and biases. Science 185, 1124–1131. 17835457 10.1126/science.185.4157.1124

[B51] Weber P. Binder K. Krauss S. (2018). Why can only 24% solve bayesian reasoning problems in natural frequencies: frequency phobia in spite of probability blindness. Front. Psychol. 9:1833. 10.3389/fpsyg.2018.01833 30369891 PMC6194348

