# Supplementary material for: Evidencing How Experience and Problem Format Affect Probabilistic Reasoning Through Interaction Analysis
Source: Front Psychol. 2019 Jul 4;10:1548. doi: 10.3389/fpsyg.2019.01548 (PMC6620894; doi:10.3389/fpsyg.2019.01548)
Supplement: Supplementary file 1 [file Data_Sheet_1.pdf]

SUPPLEMENTARY MATERIAL

Collection of Past Experience (Frequency Version)

Before starting the task, please answer these 3 questions based on your personal experience:

1. What do think, on average, the chance of a fire occurring in a school might be?

   ________________ out of ________________

2. What do you think, on average, the chance of a fire alarm going off, given that there is a real fire in the school, might be?

   ________________ out of ________________

3. What do you think, on average, the chance of a fire alarm going off, given that there isn't any real fire in the school, might be?

   ________________ out of ________________

Collection of Past Experience (Probability Version)

Before starting the task, please answer these 3 questions based on your personal experience:

1. What do think, on average, the probability of a fire occurring in a school might be?

   ________________ %

2. What do you think, on average, the probability of a fire alarm going off, given that there is a real fire in the school, might be?

   ________________ %

3. What do you think, on average, the probability of a fire alarm going off, given that there isn't any real fire in the school, might be?

   ________________ %

Textual Description (Frequency Version)

The occurrence of fire in schools is a serious issue. It can lead to the death of a large number of students and members of staff, especially if they do not adhere to the health and safety guidelines that describe how to behave in such situations. So, understanding the real risk of a fire, in the event of hearing a fire alarm, is very important. A fire alarm is not necessarily a signal of an actual fire. For instance it might be that someone is smoking in a toilet, or that the alarm goes off by accident. However, sometimes the opposite is true - i.e. there is a fire, but the alarm does not go off, perhaps because it is faulty. Suppose that you know, based on your experience, that when there is a fire most (but not all) of the times the alarm goes off. Suppose that you also know that, if there is not a fire, the alarm sometimes goes off for other reasons. Now, imagine that you are at a school on a random day of the year and you suddenly hear the fire alarm. What is the chance that there is a real fire in the school given that the alarm has gone off? To answer this question accurately you need the actual data showing the chance of fire, in the event of an alarm. This is shown in the next page using a diagram. In this diagram, the word 'events' refers to situations in which there was or there wasn't a fire, or situations in which there was or there wasn't a fire alarm sounding. We would like you to answer the question below the diagram, in the form of a proportion using positive integers. For example, you could write 1 out of 6, or 25 out of 100, or 37 out of 993. The maximum number allowed is 1000. You should base your answer on the data that is given in the diagram. In the next page you will see some boxes describing various events. These boxes cover the actual data. To access the data you should hover over the relevant box with your mouse. The data will disappear once you move out of the box. To see the data again, you need to re-hover over the box. Try to hover over and away from the box below several times to familiarize yourself with how to access the data.

Textual Description (Probability Version)

The occurrence of fire in schools is a serious issue. It can lead to the death of a large number of students and members of staff, especially if they do not adhere to the health and safety guidelines that describe how to behave in such situations. So, understanding the real risk of a fire, in the event of hearing a fire alarm, is very important. A fire alarm is not necessarily a signal of an actual fire. For instance it might be that someone is smoking in a toilet, or that the alarm goes off by accident. However, sometimes the opposite is true - i.e. there is a fire, but the alarm does not go off, perhaps because it is faulty. Suppose that you know, based on your experience, that when there is a fire most (but not all) of the times the alarm goes off. Suppose that you also know that, if there is not a fire, the alarm sometimes goes off for other reasons. Now, imagine that you are at a school on a random day of the year and you suddenly hear the fire alarm. What is the probability that there is a real fire in the school, given that the alarm has gone off? To answer this question accurately you need the actual data showing the probability of fire, in the event of an alarm. This is shown in the next page using a diagram. In the diagram, the word 'events' refers to situations in which there was or there

wasn't a fire, or situations in which there was or there wasn't a fire alarm sounding. We would like you to answer the question below the diagram, in the form of a percentage. For example, to express the probability of fire, you could write 2% or 37%, etc. You should base your answer on the probabilities that are given in the diagram. In the next page you will see some boxes describing these probabilities associated with various events - e.g., the probability of fire, or the probability of a fire alarm ringing, etc. These boxes cover the actual data. To access the data you should hover over the relevant box with your mouse. The data will disappear once you move out of the box. To see the data again, you need to re-hover over the box. Try to hover over and away from the box below several times to familiarize yourself with how to access the data.

Question for the Bayesian Problem

Frequency format: How many of the events which are associated with a fire alarm do you expect to actually signal a real fire?

________________ out of ________________

Probability format: What is the probability that there is a real fire in the school, given that the alarm has gone off?

________________ %

**Study**	**Condition**	**Correct responses**
1	Frequency	39%
1	Probability	14%
2	Frequency	9%
2	Probability	2%

Table 1: Percentages of correct answers across conditions. Results from the analysis excluding 9 observations: participants who were presented with a problem in which the hit rate was 100%).

**Factor**	**OR**	**95% CI**
Information Format	0.24	0.12-0.48
Data Generating Mode	0.14	0.07-0.28
Numeracy	1.89	0.92-3.88

Table 2: Odd ratio and 95% confidence intervals. Results from the logistics regression analysis excluding 9 observations: participants who were presented with a problem in which the hit rate was 100%).
